# Evaluation of the quality of clinical data collection for a pan-Canadian cohort of children affected by inherited metabolic diseases: lessons learned from the Canadian Inherited Metabolic Diseases Research Network

**DOI:** 10.1186/s13023-020-01358-z

**Published:** 2020-04-10

**Authors:** Kylie Tingley, Monica Lamoureux, Michael Pugliese, Michael T. Geraghty, Jonathan B. Kronick, Beth K. Potter, Doug Coyle, Kumanan Wilson, Michael Kowalski, Valerie Austin, Catherine Brunel-Guitton, Daniela Buhas, Alicia K. J. Chan, Sarah Dyack, Annette Feigenbaum, Alette Giezen, Sharan Goobie, Cheryl R. Greenberg, Shailly Jain Ghai, Michal Inbar-Feigenberg, Natalya Karp, Mariya Kozenko, Erica Langley, Matthew Lines, Julian Little, Jennifer MacKenzie, Bruno Maranda, Saadet Mercimek-Andrews, Connie Mohan, Aizeddin Mhanni, Grant Mitchell, John J. Mitchell, Laura Nagy, Melanie Napier, Amy Pender, Murray Potter, Chitra Prasad, Suzanne Ratko, Ramona Salvarinova, Andreas Schulze, Komudi Siriwardena, Neal Sondheimer, Rebecca Sparkes, Sylvia Stockler-Ipsiroglu, Yannis Trakadis, Lesley Turner, Clara Van Karnebeek, Hilary Vallance, Anthony Vandersteen, Jagdeep Walia, Ashley Wilson, Brenda J. Wilson, Andrea C. Yu, Nataliya Yuskiv, Pranesh Chakraborty, on behalf of the Canadian Inherited Metabolic Diseases Research Network

**Affiliations:** 1 University of Ottawa, Ottawa, Ontario Canada; 2 Newborn Screening Ontario, Children’s Hospital of Eastern Ontario, 401 Smyth Road, Ottawa, Ontario K1H 8L1 Canada; 3 The Hospital for Sick Children, University of Toronto, Toronto, Ontario Canada; 4 Bruyère Research Institute, Ottawa, ON Canada; 5 Department of Medicine, Ottawa Hospital Research Institute, Ottawa, ON Canada; 6 Le centre hospitalier universitaire Ste-Justine, Montreal, Quebec Canada; 7 Montreal Children’s Hospital, McGill University, Montreal, Quebec Canada; 8 Stollery Children’s Hospital, University of Alberta, Edmonton, Alberta Canada; 9 IWK Health Centre, Dalhousie University, Halifax, Nova Scotia Canada; 10 BC Children’s Hospital, University of British Columbia, Vancouver, British Columbia Canada; 11 Health Sciences Centre Winnipeg, University of Manitoba, Winnipeg, Manitoba Canada; 12 London Health Sciences Centre, Western University, London, Ontario Canada; 13 Hamilton Health Sciences Centre, McMaster University, Hamilton, Ontario Canada; 14 Le centre hospitalier universitaire Sherbrooke, Sherbrooke, Quebec Canada; 15 Alberta Children’s Hospital, University of Calgary, Calgary, Alberta Canada; 16 Janeway Children’s Hospital, Memorial University, St John’s, NL Canada; 17 Kingston General Hospital, Queen’s University, Kingston, Ontario Canada

**Keywords:** Inherited metabolic diseases, Observational research, Registry science, Data quality, Database, Sustainability

## Abstract

**Background:**

The Canadian Inherited Metabolic Diseases Research Network (CIMDRN) is a pan-Canadian practice-based research network of 14 Hereditary Metabolic Disease Treatment Centres and over 50 investigators. CIMDRN aims to develop evidence to improve health outcomes for children with inherited metabolic diseases (IMD). We describe the development of our clinical data collection platform, discuss our data quality management plan, and present the findings to date from our data quality assessment, highlighting key lessons that can serve as a resource for future clinical research initiatives relating to rare diseases.

**Methods:**

At participating centres, children born from 2006 to 2015 who were diagnosed with one of 31 targeted IMD were eligible to participate in CIMDRN’s clinical research stream. For all participants, we collected a minimum data set that includes information about demographics and diagnosis. For children with five prioritized IMD, we collected longitudinal data including interventions, clinical outcomes, and indicators of disease management. The data quality management plan included: design of user-friendly and intuitive clinical data collection forms; validation measures at point of data entry, designed to minimize data entry errors; regular communications with each CIMDRN site; and routine review of aggregate data.

**Results:**

As of June 2019, CIMDRN has enrolled 798 participants of whom 764 (96%) have complete minimum data set information. Results from our data quality assessment revealed that potential data quality issues were related to interpretation of definitions of some variables, participants who transferred care across institutions, and the organization of information within the patient charts (e.g., neuropsychological test results). Little information was missing regarding disease ascertainment and diagnosis (e.g., ascertainment method – 0% missing).

**Discussion:**

Using several data quality management strategies, we have established a comprehensive clinical database that provides information about care and outcomes for Canadian children affected by IMD. We describe quality issues and lessons for consideration in future clinical research initiatives for rare diseases, including accurately accommodating different clinic workflows and balancing comprehensiveness of data collection with available resources. Integrating data collection within clinical care, leveraging electronic medical records, and implementing core outcome sets will be essential for achieving sustainability.

## Background

Inherited metabolic diseases (IMD) are a group of more than 1000 genetic disorders that are characterized by disruptions in at least one biochemical pathway [1, 2]. Although individually rare, the overall global birth prevalence of IMD has been estimated as 50.9 per 100, 000 live births [3], representing a significant impact on population health. Advancements such as next generation sequencing, metabolomics, and newborn screening have led to earlier detection of IMD, improved understanding of the underlying biological mechanism of disease, and subsequent development of new therapeutics [4, 5]. Because of these advances in care, patients with IMD have fewer severe sequelae, in turn reducing disease morbidity and increasing life expectancy [5]. In recognition of increased life expectancy among IMD patients, a current priority in IMD research is long-term follow-up of patients to generate high quality, longitudinal clinical data to evaluate outcomes and inform both clinical care and public policy [6, 7].

While randomized controlled trials remain the gold standard for minimizing risk of bias and confounding in establishing treatment efficacy and evaluating outcomes [8], it is often not feasible to conduct a high quality clinical trial in the context of rare diseases due to an inability to recruit an adequate sample size to achieve adequate statistical power and to address the characteristic clinical heterogeneity of many rare diseases. Thus, population-based cohort studies, patient registries, and practice-based evidence networks are important tools for investigating natural history, evaluating disease management practices, establishing effectiveness of interventions, and assessing both short- and long- term outcomes [9–12]. To successfully achieve these goals, collection of high quality observational data is imperative [13, 14].

**Fig. 1:**
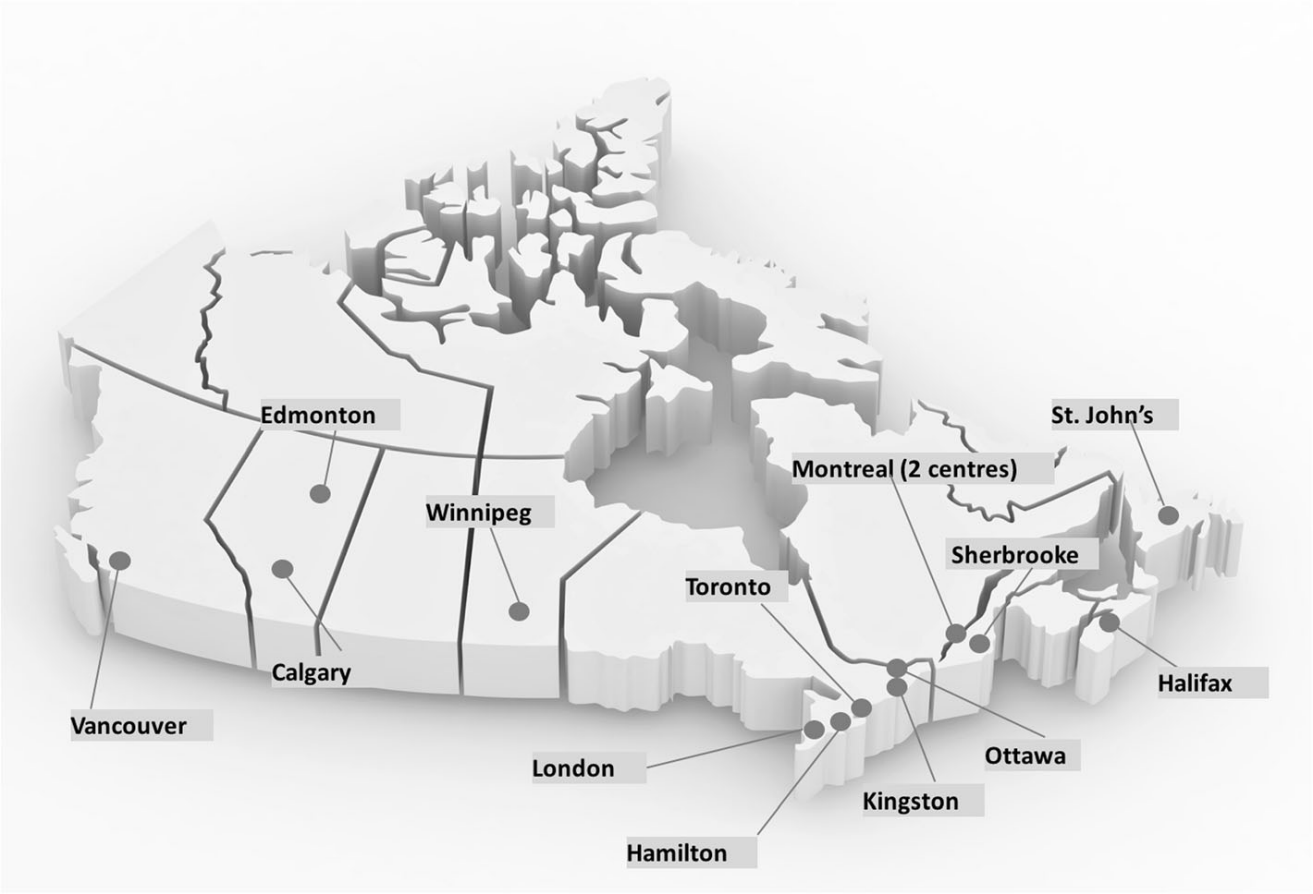
Location of Canadian Hereditary Metabolic Treatment Centres that are participating in CIMDRN (*n* = 14)

In 2012, with funding from a Canadian Institutes of Health Research Emerging Team grant, the Canadian Inherited Metabolic Diseases Research Network (CIMDRN) was established. CIMDRN is a pan-Canadian, multidisciplinary, practice-based network with the overall goal of generating high quality observational evidence to improve care and outcomes for children diagnosed with IMD in Canada [15]. Over 50 investigators from across Canada in the fields of pediatric care for IMD patients, epidemiology, and health services and policy research are involved, along with 14 Canadian Hereditary Metabolic Disease Treatment Centres (Fig. 1). CIMDRN’s *clinical research stream* uses medical chart-abstracted data to: (i) describe the longitudinal experience of a population-based cohort of Canadian children diagnosed with IMD; and (ii) investigate associations between patterns of interventions and clinical outcomes in this cohort. To achieve these goals, development of a comprehensive data quality management plan is critical to the establishment and maintenance of a high-quality clinical database.

Data quality principles established for disease registries include: completeness, accuracy, interpretability/accessibility, relevance, timeliness, coherence/comparability, and data protection/privacy [16, 17]. These principles are also applicable to our longitudinal clinical database, which, similar to a research registry, is designed to support multiple descriptive and evaluative studies. Establishing a set of quality assurance procedures (activities undertaken in advance of data collection to ensure the highest quality outputs), quality control procedures (activities undertaken during and following data collection to identify and correct errors), and quality assessment procedures (activities undertaken to evaluate the quality of the whole system) is recommended for registries to ensure robust data collection throughout all research activities [18, 19]. In this paper, we describe the design and development of our clinical data collection platform, discuss our data quality management plan, and present the findings to date from our data quality assessment, highlighting key lessons that can serve as a resource for future clinical research initiatives relating to rare diseases.

## Methods

To ensure high quality clinical data collection, we established specific data quality management strategies according to best practice guidelines for disease-specific patient registries [18, 20] and medical chart data abstraction [21, 22]. Procedures were adapted based on consultation with CIMDRN investigators and experts in database architecture and personal health information privacy.

### Developing a clinical data collection platform

We established a working group of over 20 CIMDRN investigators to advise CIMDRN’s clinical research activities. This group included care providers at each CIMDRN centre and investigators with expertise in the clinical evaluative sciences. Responsibilities of the group included: refining the list of CIMDRN-targeted IMD; developing measures of relevant clinical outcomes, interventions, and intermediate indicators of disease management; and establishing procedures for clinical data collection and implementation at each centre. A list of 31 targeted IMD was developed. We focused on treatable diseases that were characterized by important clinical and/or health policy questions. This included many target disorders of newborn screening, given the need for long-term follow-up information to better understand natural history and outcomes in a screened population (Table 1). To ensure feasibility of data collection within the resources available, we opted to identify a subset of five of CIMDRN’s targeted IMD as *priority* diseases for in-depth longitudinal data collection, based on preliminary evidence of variability in outcomes and in clinical management for these diseases (Table 1).

**Table 1:** CIMDRN-targeted IMD (*n* = 31); priority diseases (bold) have been selected by investigators for in-depth longitudinal data collection

CIMDRN targeted-IMD	
Amino acid disorders:	
**• Phenylalanine hydroxylase (PAH) deficiency**	
**•** Homocystinuria	
**•** Maple syrup urine disease	
**•** Tyrosinemia	
Urea cycle disorders:	
**•** Arginase deficiency	
**•** Argininosuccinic acidemia	
**•** Carbamyl phosphate synthetase deficiency	
**•** Citrin deficiency	
**•** Citrullinemia	
**•** Hyperornithinemia-Hyperammonemia-Homocitrullinuria syndrome	
**•** N-acetylglutamate synthetase deficiency	
**•** Ornithine transcarbamylase deficiency	
Organic acid disorders:	
**•** β-ketothiolase deficiency	
**•** Glutaric acidemia type I	
**•** HMG-CoA lyase deficiency	
**•** Isovaleric academia	
**•** 3-methylcrotonyol-CoA carboxylase deficiency (3MCC)	
**•** Methylmalonic acidemias (MMA)	
**•** Propionic acidemia	
Fatty acid oxidation disorders:	
**• Medium-chain acyl-CoA dehydrogenase (MCAD) deficiency**	
**• Very long-chain acyl-CoA dehydrogenase (VLCAD) deficiency**	
**•** Carnitine uptake defect (CUD)	
**•** Long-chain 3-hydroxyacyl-CoA dehydrogenase deficiency	
**•** Trifunctional protein deficiency	
Other disorders:	
• Guanidinoacetate methyltransferase (GAMT) deficiency*	
• Mucopolysaccharidosis type I (MPSI)*	
**•** Farber disease*	
**•** Galactosemia	
**•** Glycogen storage disease type I	
**•** Multiple carboxylase deficiency/biotinidase deficiency	
**•** Pyridoxine-dependent epilepsy	

*individuals of any age enrolled if receiving care at a participating Centre

Through consensus of the working group, we established a framework (Table 2) to guide the development of a set of data elements with operationalized definitions that would be collected systematically from participants’ medical charts. Specific data elements were chosen by the working group based on their perceived clinical importance and consideration of whether the information was likely to be present in participants’ medical record. We also considered inclusion of data elements from related longitudinal research projects, for example, the Newborn Screening Translational Research Network (https://www.nbstrn.org/) [23] and the Urea Cycle Disorders Consortium (https://www.rarediseasesnetwork.org/cms/ucdc) [24].

**Table 2:** Framework used to guide the choice and definitions for data elements included in our clinical data collection tool

	Demographics	Diagnosis	Clinical descriptors	Secondary diagnoses	Interventions	Covariates	Outcomes
**Description**	General patient information	Definition should be broad to reflect clinical heterogeneity and be as inclusive as possible of individuals with possible health issues related to this IMD	Variables informing diagnosis, tissue involvement, severity, and pathophysiology related to primary diagnosis and/or other acute/chronic diagnoses	Diagnoses resulting from complications of the primary diagnosis or related interventions, as well as apparently unrelated acute or chronic diagnoses	Exposures that are manipulated by care providers to change natural history	Other factors postulated to influence the outcome(s)/natural history	Variables reflecting the health and functional status of the patient, including patient/family-centred variables

### Quality assurance

#### Eligibility criteria

The same eligibility criteria were applied across participating sites. Children were enrolled (with informed, parental consent and child assent, if applicable) in CIMDRN’s clinical research stream if they were:

- diagnosed with one of the targeted IMD
- born from 2006 to 2015 (any age for 3 ultra-rare diseases, see Table 1)
- received care at one of 14 participating Canadian Hereditary Metabolic Disease Treatment Centres

#### Centralized data collection

Centre staff reviewed participants’ medical records and securely recorded information in our centralized clinical database, facilitated by Research Electronic Data Capture (REDCap) and hosted at the Children’s Hospital of Eastern Ontario Research Institute. REDCap is a secure, web-based application that is designed to support data collection for research studies [25]. At some sites, the participants’ metabolic clinic information was accessed via their hospital electronic medical record, while at other sites without electronic medical record infrastructure, information was accessed via their paper chart held within the metabolic clinic. Research staff at each satellite site were given a data entry training session via teleconference/webinar by staff at the central coordinating site and were provided with a comprehensive data entry manual and frequently-asked-questions document to guide them through data entry.

**Fig. 2:**
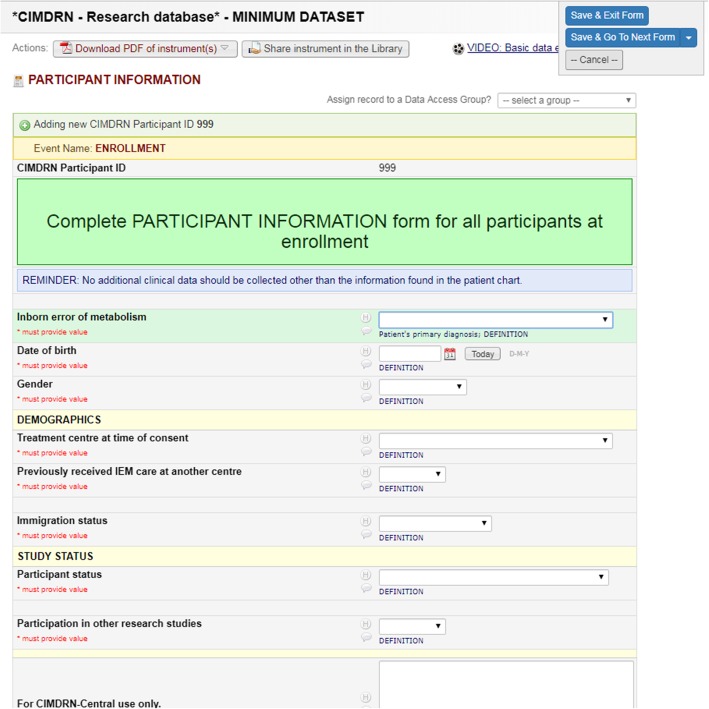
Example data collection form in Research Electronic Data Capture (REDCap) [25]

Given that staff extracting data from medical charts at participating centres had differing professional backgrounds (e.g., dietitians, clinical research coordinators, fellows/ trainees, research students) and not all were content experts in the field of IMD, an intuitive and user-friendly interface (Fig. 2) for data entry was critical for quality assurance (data collection tools available upon reasonable request). We organized data fields into a set of short forms, similar to a series of web surveys, by type of information collected (i.e., participant characteristics, family/household information, diagnostic laboratory investigations, monitoring laboratory tests, ongoing clinic visits). We also used branching logic to organize data fields according to the participant’s diagnosis (e.g., routine laboratory test options specific to monitoring medium-chain acyl-CoA dehydrogenase (MCAD) deficiency are not displayed for a participant diagnosed with a different IMD).

We designed our clinical data collection forms to limit the potential for missing data and data entry errors. When possible, our data fields relied on close-ended responses (e.g., drop down menus with multiple choice or check-all-that-apply response options) that included an “unknown” or “not applicable” option in case that information had not been recorded in the chart, or an “other, please specify” category in case the applicable option was not readily available. Additionally, for data fields that were open-ended, we required that any empty fields be “verified as missing” (i.e., confirmation by the individual entering data that the information was not recorded in the chart); this was facilitated using a built-in data verification tool in REDCap. REDCap also allows for some automated validation of data fields (e.g., ensuring that a recorded value/date is within a pre-defined range or ensuring that only numeric characters can be entered in a numeric field). We also set automated calculations for derived variables, such as body mass index (based on entered height and weight), to protect against errors in calculations entered manually.

For all 31 targeted IMD, we collected a minimum set of data that included information on sociodemographic characteristics, case ascertainment and diagnosis, and initial interventions. For children with the five IMD we prioritized for in-depth longitudinal data collection, we collected information beyond the diagnostic period, which was organized around each follow-up visit to the metabolic clinic. These data included information about interventions received, clinical outcomes, and indicators of disease management. Depending on branching logic, there were between 37 and 63 required data fields for each disease in the minimum dataset, and between 39 and 68 required data fields at each clinic visit for longitudinal data collection. Each database also contained several hundred additional data fields that depend on the specific disease and branching logic options (e.g., specific laboratory test results, prescriptions, etc.). To limit misinterpretation of data fields, we added a definition developed by the clinical research working group to each data field and incorporated the definitions into a data dictionary that was made available to participating centres through REDCap.

This study was reviewed and approved by the research ethics board at each participating centre (see Acknowledgements).

#### Data privacy/protection

We developed a set of data privacy procedures that were agreed upon among the participating sites. Specifically, as described above, all data were collected using REDCap and are stored within the Children’s Hospital of Eastern Ontario Research Institute secure server(s). REDCap uses a 128-bit data encryption and requires a user ID and password to access [25]. To protect participant identification and privacy, we generated study-specific ID numbers for each participant and did not collect any overtly identifying information within the clinical research database (a separately partitioned database stores contact information for participants from selected centres who agree to be contacted to participate in future research). In addition, CIMDRN investigators and staff at each satellite site are only able to view clinical data for the participants enrolled at their centre. Investigators and staff at the central coordinating site are able to access clinical data for participants across all sites in order to facilitate centralized quality control activities (see below) and conduct analyses. We have developed a data request policy that allows any CIMDRN investigator to request de-identified data for participants across all sites provided their request is approved by CIMDRN’s Data Advisory Committee. Finally, we do not publicly report potentially identifying results for any cell with fewer than five participants contributing data.

### Quality control

#### Communication with satellite sites

Routine communication between CIMDRN’s central office and each satellite site was a key component for quality control. Teleconferences were held with the site coordinators 1–2 times per year to discuss and answer questions about clinical data collection and other CIMDRN-related activities. The central office also maintained and updated a data entry manual and an electronic frequently-asked-questions document, accessible via REDCap. This document included questions and answers reflecting concerns raised by satellite sites as data collection progressed. A bulletin was also sent from the central office via e-mail to site coordinators every 2–4 weeks, highlighting any improvements and/or changes made to the clinical database (e.g., addition/removal of data fields). The CIMDRN central office also regularly issued individual status reports to each of the sites, which provided specific feedback to each centre about their data entry progress and highlighted any potential data quality issues noted by the central office. Finally, site coordinators were encouraged to contact the central office with any questions or concerns about data collection. Central site staff responded within 1–3 days and arranged telephone or webinar support as appropriate. In response to feedback received from our satellite sites, we occasionally made improvements to our data collection forms, and maintained a change management log to track these changes.

#### Centralized data verification

Each participant’s data were subject to centralized verification using REDCap-enabled correspondence between the central office and each satellite site to address specific concerns. See Table 3 for an overview of items that were checked as part of this verification process. Once the central office was satisfied that data had been entered correctly for a given participating individual, the data collection forms for that individual were marked as ‘verified’, locked to prevent any changes without approval by the central site, and associated data were considered useable for analysis.

**Table 3:** Data validation items developed by the central CIMDRN office

Items verified	
**•** Check that blank data fields are confirmed as intentionally blank to indicate that information is not available in the participant’s chart	
**•** Check that any dates that are entered occur within a reasonable time frame (e.g., dates should not occur in the future)	
**•** Check that participant data are recorded in chronological order	
**•** Follow up with centre regarding any extreme values that could represent a data entry error	
**•** Check to ensure correct organization of information (i.e., data entered in correct data field and in the correct format)	
**•** Check that organization of data in clinical database follows expected workflow at that centre (i.e., order of events is reasonable and appropriate)	
**•** Ensure appropriate unit conversion, if applicable	

### Data quality assessment

The central office also conducted routine monitoring of aggregate data (descriptive analysis) to identify the frequency of item-missing data, both overall and by centre, and to identify systematic differences in data elements across centres. When we identified systematic differences or unusual data patterns, we followed up with centre staff to determine whether data fields had been correctly interpreted and whether there were circumstances at a centre (e.g., charting practices) that may have compromised the availability or quality of data. We also communicated preliminary descriptive findings from participants enrolled across sites to clinical investigators in the network, to discuss potential sources of variation, which may reflect real differences in practice or data quality. Below are the results from our data quality assessment to date, including the proportion of data entry that is complete, frequency of missing data, and discussion of other potential data quality issues that were identified during our assessment.

## Results

### Data entry

**Fig. 3:**
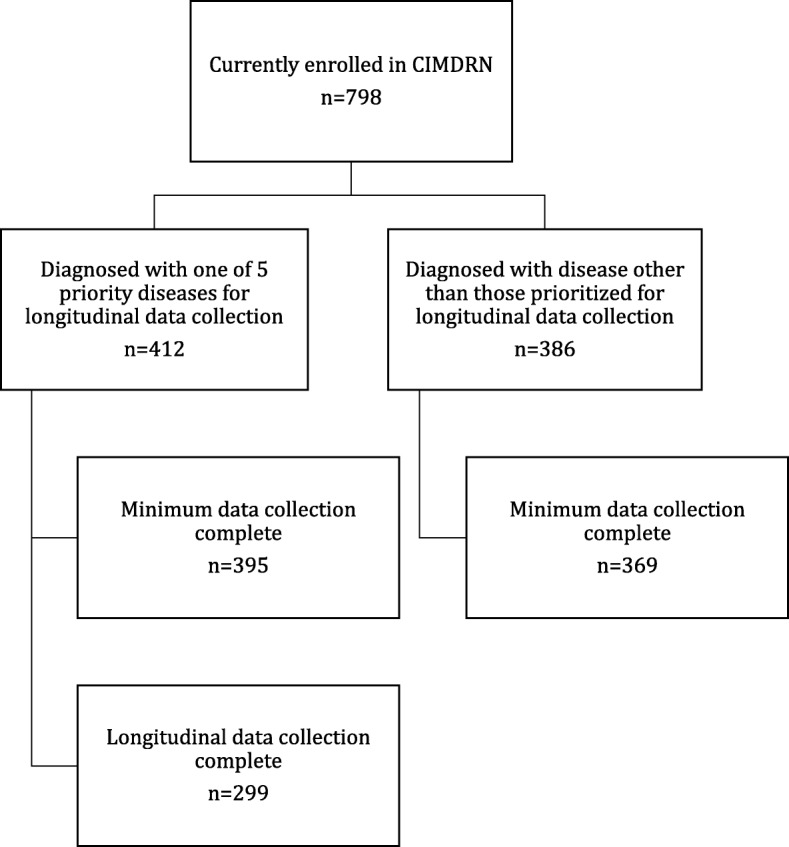
CIMDRN participant flow diagram as of September 24, 2019

We have enrolled 798 children in CIMDRN, of whom 412 (52%) have been diagnosed with one of the five diseases prioritized for longitudinal data collection. Data entry (minimum and longitudinal datasets) is underway or complete at 13 of the participating centres (one centre has yet to begin patient enrollment). Of the 798 participants currently enrolled in CIMDRN, 764 (96%) have complete minimum dataset information recorded in the clinical database (Fig. 3). Among the 412 participants diagnosed with a priority disease, 299 (73%) have complete longitudinal follow-up data recorded in the clinical database (Fig. 3).

### Data quality assessment

To date (September 24, 2019), our data quality assessment has prioritized the minimum dataset (sociodemographic information, ascertainment and diagnosis information) for all CIMDRN-targeted IMD and the longitudinal dataset information (interventions received, clinical outcomes, indicators of disease management) for phenylalanine hydroxylase (PAH) deficiency.

#### Item-missing data

As expected, among the 743 participants with complete minimum dataset information, missing data were common for many sociodemographic variables due to information not being reported in participants’ medical charts at some or all centres. For example, guardian’s employment status was missing for over 50% of participants, and number of people living in a participant’s primary household was missing for 27% of participants. Little to no information was missing regarding disease ascertainment (e.g., by newborn screening, clinical symptoms, and/or family history – 0% missing) and diagnostic timelines (9% missing diagnosis date/age, 9% missing newborn screen positive date, Table 4). For diagnostic tests considered universally important, little information was missing from participants’ medical charts (Table 4).

**Table 4:** Missing data for select ascertainment and diagnosis among participants with complete minimum dataset information (*n* = 764)

IMD	Ascertainment and diagnostic workup variables	% missing
Across all CIMDRN-targeted diseases	Ascertainment method (e.g., by NBS, family history, etc.)	0%
	Number of visits to metabolic clinic to determine diagnosis	9%
	Age at diagnosis	9%
	Centre where diagnosis was established	0%
	For those not ascertained by NBS (*n* = 138), was a NBS test done?^a^	50%
	For those ascertained by NBS (*n* = 626), was the NBS test positive for the diagnosed disease?^a^	1%
	For those whose NBS test was positive (*n* = 620), date of NBS test positive/referral^a^	9%
	For those diagnosed symptomatically (*n* = 125), age at first symptom^a^	17%
	For those diagnosed symptomatically (n = 125), presenting symptoms^a^	0%
**Diagnostic tests considered universally important**		
PAH deficiency (*n* = 215)	Plasma amino acid profile	2%
MCAD deficiency (*n* = 127)	Acylcarnitine profile	10%
VLCAD deficiency (*n* = 33)	Acylcarnitine profile	< 10%
MPS type I (*n* = 18)	α-L-iduronidase activity	< 10%

*IMD* inherited metabolic disease, *NBS* newborn screening, *PAH* phenylalanine hydroxylase, *MCAD* medium-chain acyl CoA dehydrogenase, *VLCAD* very long-chain acyl-CoA dehydrogenase, *MPS* mucopolysaccharidosis

^a^denominators for these data elements vary as not all variables were applicable to every participant

In cases where important information regarding ascertainment and diagnosis appeared to be missing, we contacted the research staff at the corresponding site to try to determine the specific reasons for these missing data. In many of these cases, missing information was due to participants moving or transferring care. For example, 13 of 127 participants diagnosed with MCAD deficiency (10%) did not have an acylcarnitine profile recorded in the diagnostic test data available to CIMDRN (Table 4). We have determined that among these 13 participants, most were not diagnosed at their consenting centre and did not have diagnostic test results transferred to their current centre. Other reasons for missing data on this variable included early mortality and diagnosis confirmed by testing for a familial mutation.

Missing data were, at times, related to the organization of patient charts. For example, while 113 individual neuropsychological assessments were recorded among 22 PAH deficiency participants, only 84 (74%) of those assessments were accompanied by test results in participants’ main hospital charts (i.e., in the other cases, the chart indicated that the test was ordered or completed, but the test-specific results were not present). For example, at one centre, 100% of the neuropsychological tests recorded in our database have accompanying results, compared to 37% of those for participants from another centre. Upon further investigation, we learned that at some centres, neuropsychological test results are not routinely filed into the patient’s main hospital chart accessible at the metabolic clinic and therefore for data entry for our cohort.

#### Other data quality issues

During the design phase, we recognized that participants may move among participating treatment centres during the study period and, consequently, may be enrolled at more than one centre. We added a variable to our database about previous receipt of care at another centre and have proactively reviewed participants’ date of birth and diagnosed IMD to ensure that potential duplicate participants would be identified. If a participant was identified as a potential duplicate, we contacted the appropriate treatment centres to confirm. Using this strategy, we have identified three participants who were enrolled at more than one centre and we can link their clinical data as appropriate for specific analyses.

Identification of extreme values via routine descriptive analyses has also prompted further evaluation of some participants’ individual data. As an example, age at diagnosis for participants with PAH deficiency initially ranged from 2 to > 200 days, which led to a detailed review of the data for participants diagnosed beyond the first month of life to ensure the accuracy of data. Upon further investigation, we determined that differing interpretations of the definition for age at diagnosis (i.e., “date upon which investigation results confirming the diagnosis become available”) among the treatment centres were contributing to the inconsistencies. In particular, some treatment centres assigned a definitive diagnosis based on test results that take longer to perform (e.g., molecular genetic testing) than others (e.g., plasma amino acid profile) and yet the corresponding participants were fully treated under a presumptive diagnosis while waiting for these final test results. Similarly, clarification about definitions for other variables (e.g., date of a positive newborn screening test referral, modified medical foods, clinic visit) have been necessary in response to queries or due to systematic variation across centres. For example, one participating centre routinely conducts a second newborn screening test as part of their diagnostic workup for some patients, prompting clarification about which date to use as the newborn screen positive referral date. For some centres, telemedicine is commonly used to provide care to patients living in rural or remote areas of Canada so further explanation about the definition of a clinic visit was necessary to ensure that telemedicine information was being captured appropriately in our clinical database. To ensure the reliability of results generated from our clinical data analyses, we have chosen to minimize the use of variables for which we have determined inconsistent interpretations among participating centres in our analyses. The data dictionary used for our study is available upon reasonable request.

## Discussion

### Lessons learned

Reliable clinical data collection is crucial in generating robust evidence about the natural history, management strategies, and clinical intervention effectiveness for IMD. Incomplete and/or inaccurate data collection compromises the interpretation of results; therefore, it is important to have methods in place to identify and mitigate any potential issues with data completeness or accuracy [19, 20]. Throughout the design, implementation, and maintenance of our clinical data collection platform, we have learned several lessons that can serve as a resource for future clinical research initiatives relating to rare diseases. Consideration of these issues is important for the long-term sustainability of in-depth, longitudinal clinical data collection initiatives such as ours.

#### Lesson learned #1: mitigating potential data quality issues relies on good communication with participating sites and centralized data validation measures

One of the main factors contributing to our success has been maintaining close communication between the satellite sites and the central team. With a relatively small number of participants, we were able to directly correspond with research staff at each centre to resolve many data quality issues and improve the quality of our data virtually in real-time, as the data were entered. Comparable rare disease initiatives have reported using similar strategies, such as having a central coordinating office monitoring data collection and regularly querying participating satellite sites when data entry errors are suspected [26, 27]. In addition, engaging with clinician investigators when reporting preliminary findings helped to distinguish potential data quality issues from areas where there is true practice variation. The research staff who abstracted data from patient charts at most centres had expertise or familiarity with IMD patient care, which further helped by minimizing the potential for misinterpretation of information.

We have also been successful in mitigating potential data quality issues, including item-missing data, by using a variety of centralized data validation measures. Built-in automatic data validation and centralized manual data verification were both important for the maintenance of data collection and have also been reported by others as strategies for avoiding data entry errors [26–28]. We did not formally assess inter-rater reliability with respect to data entry at each site because of limited resources; future endeavours should consider building such reliability assessments into the data quality plan.

#### Lesson learned #2: to limit misinterpretation of data elements during data collection, data collection tools must accommodate different clinic workflows and provide clear definitions for each data element. The use/analysis of data elements with known inconsistencies in their interpretation should be limited

Despite efforts to ensure from the outset that our clinical data collection platform could be easily translated across treatment centres, challenges remained in accurately accommodating differences in workflow or charting practices. For example, the type of information and the level of detail captured within participants’ metabolic medical record was not always consistent among centres. In response, we added new data elements or modified existing data elements as needed (e.g., adding telemedicine variables to account for diet changes that occur over the phone or by email between participants and their dietician, adding more response options for laboratory locations). With incremental enrollment and data entry over time, making these post hoc changes to our database while ensuring consistency with previously-entered data was relatively straightforward, although it required a commitment at the satellite centres to occasionally re-visit patient charts and make changes for consistency. Similarly, we have noted different interpretations of specific data fields among centres despite attempts to provide a clear and comprehensive definition for each data element (e.g., modified medical food). Where possible, we have further clarified these definitions either directly in the database and accompanying data dictionary or through communications with participating centres, and have highlighted and corrected potential erroneous data.

#### Lessons learned #3: ensure that data collection is feasible among participating sites by balancing volume of data being recorded with available resources

Developing, implementing, and maintaining a multi-centre, clinical data collection platform is resource-intensive. Balancing comprehensiveness and feasibility of data collection was difficult. This is especially true in the context of rare diseases where there is often substantial uncertainty around which elements of patient care are most important to collect. We sought to be exhaustive in our data collection effort and record hundreds of data elements per participant; however, this likely impacted the timeliness of data collection among the satellite sites and may have compromised the completeness of our dataset.

#### Lessons learned 4: ensure that each participating site’s information system will be compatible with chosen data collection system/software

Limitations with the information technology infrastructure across centres also impacted the timeliness of data collection. For example, some institutions faced difficulty with the compatibility and operation of REDCap on their hospital information system. The initial version of REDCap had slow loading times for the database because of the large number of data elements in our project; however, an update to the REDCap software and some reorganization of our clinical data to lessen the amount of data being loaded per page resolved this within several weeks.

### Sustainability

Similar to a research registry, our clinical data collection platform was designed to support multiple descriptive and evaluative studies. While we have successfully enrolled nearly 800 participants in our cohort and have collected a comprehensive set of clinical data, sustainability of this initiative is challenged by the intensity of resources required. Towards developing a more sustainable strategy for long-term clinical data collection for pediatric IMD in Canada, we are currently developing a strategy to evolve our clinical database into a high quality disease registry that may be used as a platform to launch registry-based randomized trials [29, 30], as well as contribute to better understanding the natural history of targeted IMD [20]. Developing a disease registry whose purpose is to collect high quality data to support on-going descriptive and evaluative studies offers several advantages in the context of rare diseases, including: i) improved efficiency for recruiting participants into other studies; ii) increased external validity of study results as inclusion criteria are typically broad; iii) opportunity to answer questions regarding comparative effectiveness of interventions in real-world settings; and iv) potential to collect data and better understand long-term outcomes. In order to achieve the goal of a sustainable disease registry, we will need to streamline our data collection. The use of core outcome sets [31] and/or integration with electronic medical records could facilitate high quality data collection into a disease registry. Additionally, the use of standardized vocabulary, such as the Human Phenotype Ontology [32], for data elements would improve the interoperability with other registries/data sources [19].

## Conclusions

Successfully establishing and maintaining a clinical data collection platform for a population-based cohort of children affected by IMD has been both exciting and challenging. The data collected as part of CIMDRN’s clinical research stream is ideally suited for better understanding natural history of the targeted IMD, evaluating disease management practices, establishing effectiveness of interventions, and assessing both short- and long- term outcomes. The experiences from this project will be used as scaffolding toward a more sustainable strategy for long-term clinical data collection for pediatric IMD in Canada via development of a high quality disease registry. Integrating data collection within clinical care, leveraging electronic medical records, and implementing core outcome sets will be essential for achieving sustainability.

## Data Availability

The datasets generated and/or analysed during the current study are not publicly available in order to protect the privacy of the study participants. Other materials used for this study (e.g., data collection tools, data dictionary, detailed data privacy/protection procedures, etc.) are available from the authors upon reasonable request.

## Abbreviations

CIMDRN: Canadian Inherited Metabolic Diseases Research Network
IMD: Inherited metabolic disease
MCAD: Medium chain acyl-CoA dehydrogenase
NBS: Newborn screening
PAH: Phenylalanine hydroxylase
